# Adult-Onset Still’s Disease following Coronavirus 2 (SARS-CoV-2) Vaccination: A Case Report

**DOI:** 10.3390/vaccines10101687

**Published:** 2022-10-09

**Authors:** Xiang-He Chua, Wea-Lung Lin, Yuan-Ti Lee

**Affiliations:** 1 School of Medicine, Chung Shan Medical University, Taichung City 40201, Taiwan; 2 Department of General Medicine, Kaohsiung Medical University Hospital, Kaohsiung Medical University, Kaohsiung City 80708, Taiwan; 3 Department of Pathology, Chung Shan Medical Hospital, Taichung City 40201, Taiwan; 4 Division of Infectious Diseases, Department of Internal Medicine, Chung Shan Medical University Hospital, Taichung City 40201, Taiwan

**Keywords:** adult-onset Still’s disease, COVID-19 vaccines, mRNA COVID-19 vaccine

## Abstract

In recent years, during the ravages of COVID-19, a variety of vaccines have been developed and are now on the market. However, although these new vaccines have undergone various trials, there are still many unknown side effects. We report a case of a 30-year-old woman who presented with general weakness, sore throat, generalized skin rashes, symmetrical arthralgia, and persistent fever of up to 40 °C with onset 16 days after receiving the Moderna COVID-19 vaccine. Adult-onset Still’s disease (AOSD) was diagnosed according to Yamaguchi’s criteria after excluding the feasibility of infectious diseases, autoimmune diseases, and malignancies. In particular, her responses to glucocorticoids and naproxen were significant and inversely proportional to her use of empirical antibiotics in the initial stage of treatment. We studied some similar cases of AOSD, which also considered the adverse effects of COVID-19 vaccination and suggested the immunogenicity and possibility of inflammatory responses related to COVID-19 vaccination.

## 1. Introduction

Severe acute respiratory syndrome coronavirus-2 (SARS-CoV-2) has caused a global catastrophe since the first case was reported in Wuhan in 2019. In these years, the death rate has continually risen to dire numbers. With the promulgation of various isolation policies, the global economy has also been hit hard as never before. Today, the global infection rate has reached 613 million, and the death rate has reached 6.51 million [1]. To prevent the situation from deteriorating, hundreds of vaccines have been invented. Two dozen of these vaccines have also been evaluated in phase III clinical trials. Various types of adverse effects have been reported since the popularization of vaccination. The symptoms reported as adverse effects were mostly mild to moderate, such as pain, swelling, redness at the vaccine site, fever, fatigue, headache, chills, vomiting, arthralgia, myalgia, and urticaria [2]. Regarding mRNA vaccines, the main adverse effects reported among adults were anaphylaxis, pericarditis, and myocarditis [3]. Due to its rarity, we would like to report a case of adult-onset Still’s disease (AOSD) after mRNA vaccination. Initially, we collected two suspected AOSD cases related to an mRNA vaccine for COVID-19 (Moderna). We then excluded one of them, as it was found to be a consequence of Legionella infection. However, we learned that Legionella infection might mimic the symptoms and signs of AOSD related to COVID-19 vaccination, and the diagnostic process of AOSD following COVID-19 vaccination will be described in detail below. 

## 2. Case Description

The first case was a 30-year-old woman who accepted her first dose of the Moderna COVID-19 vaccine on 31 August and her second dose on 26 October 2021. She was suffering from general weakness, sore throat, and generalized skin rashes without an itch followed by a persistent fever of up to 40 °C with chills approximately two weeks after her second dose of vaccine. She had been visiting local clinics, but the situation was becoming worse. As the disease progressed, multiple arthralgias were found, and she was then transferred to our medical center. 

**Figure 1:**
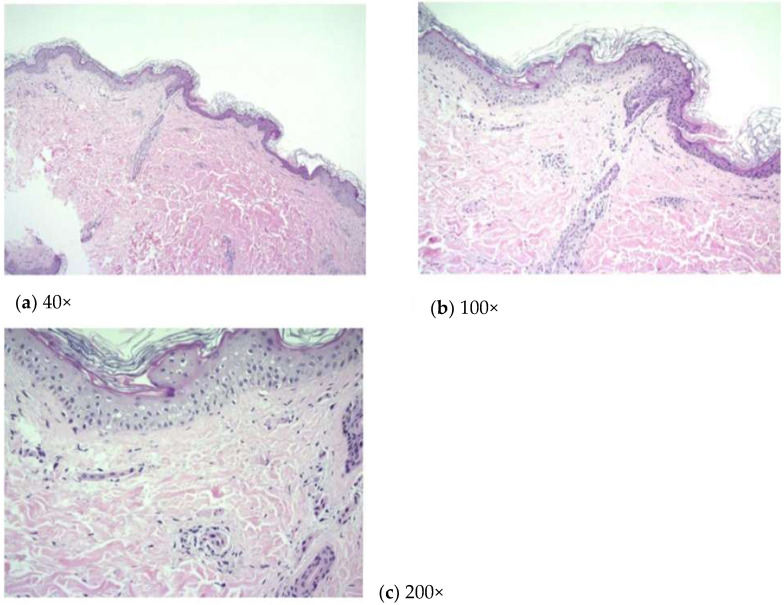
(**a**) 40×; (**b**) 100×; (**c**) 200×. Mild lymphoplasmacytic infiltrations were observed in the perivascular area.

Upon admission, she had daily fevers up to 40 °C; generalized blanchable salmon-colored edematous patches over the trunk and four limbs and also on the right palm, but not the face, without an itch; and arthralgia. The initial laboratory test revealed leukocytosis with neutrophil predominance, elevated aspartate aminotransferase (AST), and alanine aminotransferase (ALT) without significant infection. We treated her with empirical antibiotics but without success. An extensive infection survey was carried out during admission while Epstein–Barr virus (EBV) infection was ruled out, although EBV viral capsid antigen IgM and IgG were detected due to a non-detectable viral load of EBV. A polymerase chain reaction (PCR) test for COVID-19 infection had been administered upon her admission, and the result was negative. Consequently, we surveyed the autoimmune profile, and the antinuclear antibody was found to be positive (1:80×), whereas other autoimmune biomarkers were all negative. Laboratory results were as follows: white blood cell counts, 16.95 × 10^3^ cells/μL with 84.5% neutrophils; high-sensitivity C-reactive protein levels (Hs-CRP), 27.695 mg/dL; ferritin concentrations, 7675 ng/mL; aspartate aminotransferase, 66 IU/l; and alanine aminotransferase, 103 IU/l. Given the persistent remaining generalized skin rashes, combined care was arranged with a dermatologist, and a skin biopsy was ordered. The pathological report showed mild lymphoplasmacytic infiltrations in the perivascular area (Figure 1).

**Figure 2:**
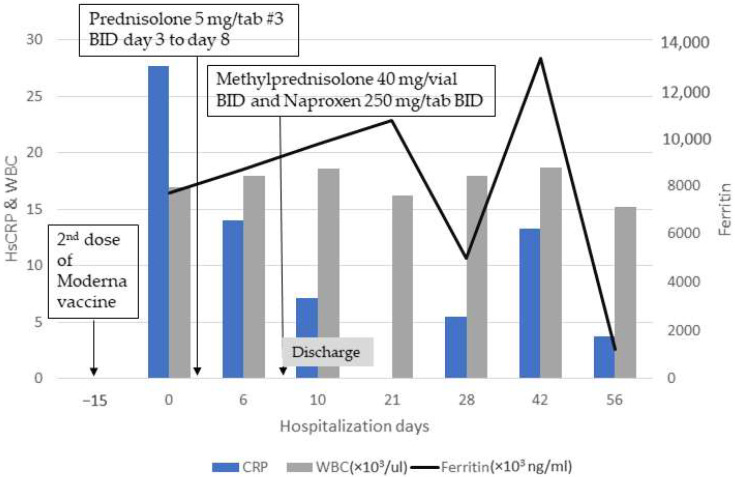
Clinical course and treatment of patient who received the Moderna COVID-19 vaccine. This patient had been suffering from general weakness, sore throat, and generalized skin rashes without an itch followed by a persistent fever of up to 40 °C with chills approximately two weeks after her second dose of the Moderna COVID-19 vaccine. Prednisolone 15 mg twice daily was administered, but improvement was limited. Her clinical condition improved significantly, and HsCRP decreased steadily after we switched her medication to methylprednisolone 40 mg intravenously twice a day and naproxen 250 mg twice a day. Hs-CRP, high-sensitivity C-reactive protein; WBC, white blood cells.

We confirmed AOSD diagnosis, as the patient was eligible according to the Yamaguchi criteria [4] by meeting the four major criteria (fever, typical rash, arthralgia, and leukocytosis) and two of five minor criteria (sore throat and abnormal aminotransferase). After diagnosis, she was initially treated with prednisolone 15 mg twice daily, but improvement was limited. We changed her medication to methylprednisolone 40 mg intravenously twice daily and 250 mg of naproxen twice daily, and a resolution was observed immediately thereafter (Figure 2). She was discharged after achieving stable clinical condition, and we have been tracking her condition at our outpatient department. During follow-up, a fluctuation in ferritin concentrations was observed, but fortunately, her disease was in complete remission after 7 months of follow up.

## 3. Discussion

Adult-onset Still’s disease (AOSD) is a rare systemic inflammatory disorder that is primarily characterized by quotidian fever, evanescent salmon-colored skin rashes, arthritis, and leukocytosis with neutrophil predominance. The majority of patients might experience myalgia, lymphadenopathy, and splenomegaly while some patients experience hepatomegaly, pleurisy, pericarditis, and abdominal pain. Macrophage activation syndrome is an infrequent but potentially fatal complication of AOSD. We understand that AOSD is a diagnosis of exclusion; therefore, the feasibility of infectious diseases, autoimmune diseases, and malignancies was excluded prior to the diagnosis of AOSD. However, the pathway linking AOSD and COVID-19 vaccination requires further study, and similar cases need to be reviewed to determine the possible mechanism. Therefore, we compared a few similar cases diagnosed as AOSD as a consequence of COVID-19 vaccination [5,6,7,8,9,10] (Table 1).

The etiology of AOSD is currently unclear, but the immunity pathogenesis of AOSD is believed to be induced by genetic susceptibility and environmental triggers such as pathogen-associated molecular patterns (PAMPs) and danger-associated molecular patterns (DAMPs). This further induces the activation of macrophages and NLRP 3 inflammasomes. This inflammasome promotes the activation of caspase 1, which leads to the proteolytic cleavage of interleukin (IL)-1β and IL-18. The bioactive form of both interleukins might produce a cytokine storm that includes IL-6, IL-8, and tumor necrosis factor (TNF) α. These cytokines and the activated form of macrophages stimulate the release of ferritin, which plays an important role in inflammatory progress [11,12]. Interestingly, COVID-19 infection shares some similarities with the pathogenesis of AOSD [13]. The immune system is alarmed by the cellular invasion of the virus. The recruitment of T helper cells can induce a cytokine storm that includes IL-6-, interferon γ-, and interferon γ -induced protein. The interferon γ- and interferon γ-induced proteins are responsible for a paracrine effect that signals viral defense. Meanwhile, chemokines such as IL-6 activate neutrophils to enhance virus engulfment [14]. A systematic review suggested that the serum level of IL-6 was elevated in patients with COVID-19 infection and that the serum level of IL-6 was proportional to the severity of the disease [15]. Another study suggested that the release of excessive levels of ferritin was not only observed in AOSD but also in COVID-19 infection: so-called “hyperferritinemic syndrome” [13]. Therefore, it is extremely important to exclude the possibility of infection before further diagnostic work is performed. The PCR test was performed in our patient to rule out COVID-19 infection, and, fortunately, the result of the test was negative. 

There is a limitation in our study because there is still a lack of evidence regarding an adverse reaction caused by the vaccine. Additionally, the possibility of the flare-up of existing AOSD should not be excluded in this group of patients. A series of case reports has shown that the administration of the COVID-19 vaccine could induce a robust immune response [16,17,18,19,20]. Existing autoimmune diseases might relapse due to the robust immune response triggered by the administration of the vaccine. Thus, we were unable to distinguish whether an adverse effect of vaccination was the induction of newly onset AOSD or whether it was actually a flare-up of existing AOSD that plays a major role in this mechanism. More studies and case reviews were needed to understand the detailed mechanism and to review the suitability of using the same diagnostic criteria under different circumstances. 

According to data from the European Medicines Agency, we affirmed that immune-related diseases account for a certain proportion of the adverse effects caused the Moderna vaccine [21]. The administration of a vaccine might further stimulate autoimmunity. Molecular mimicry is believed to be one of the proposed mechanisms of vaccine-induced autoimmunity. However, the details of the mechanism are not fully understood. In particular, there is an interesting phenomenon that shows a low incidence rate of reporting AOSD as an adverse event to the responsible government department, but several case reports related to AOSD following vaccination against COVID-19 have been published in the literature. This raises the suspicion that perhaps AOSD following the vaccine is vastly underestimated. Clinicians should be more aware of these sequelae of COVID-19 vaccination, which could continue in the future. 

## 4. Conclusions

In conclusion, the adverse effect of COVID-19 vaccination causing AOSD can be found as a rare coincidence [5]. The relationship between vaccine-induced autoimmunity, newly onset AOSD, and the relapse of existing AOSD is currently unclear. It should be remembered that exclusion of COVID-19 infection was listed as an exclusion criterion in the diagnostic criteria of AOSD due to similarities in the pathogenicity. More studies and case reviews are needed to understand the entire mechanism of vaccine-induced autoimmunity. Going back to our cases, we gained experience in diagnosing AOSD following COVID-19 vaccination, and this experience has alerted us to be more rigorous.

## Figures and Tables

**Table 1:** Comparison of various cases reported in the literature. Several reported cases were studied to compare similarities and differences. The details of cases such as the age of onset, onset after vaccination, types of vaccine received, symptoms, diagnostic criteria used, and treatment are listed above; meanwhile, every symptom of the case above was summarized into a list for a clearer comparison.

	Newly Onset Adult-Onset Still’s Disease-like Syndrome after ChAdOx1 nCoV-19 Vaccination—A Case Series with Review of Literature [5]	Adult-Onset Still’s Disease after BNT162b2 mRNA COVID-19 Vaccine [6]	Adult-Onset Still’s Disease after mRNA COVID-19 Vaccine [7]	Present Patient
Age of onset (years) and sex	Case 1: 20 (female)	36 (female)	45 (female)	30 (female)
	Case 2: 47 (female)			
	Case 3: 35 (female)			
Onset after vaccination	Case 1: 10 days (1st dose)	10 days (1st dose)	5 days (2nd dose)	16 days (2nd dose)
	Case 2: 3 weeks			
	Case 3: 3 months (1st dose)			
Vaccine	ChAdOx1 nCoV-19	BNT162b2 mRNA COVID-19 vaccine (Pfizer)	Moderna (mRNA vaccine)	Moderna (mRNA vaccine)
Symptoms	Case 1: ■ Fever (biquotidian) ■ Sore throat ■ Myalgia ■ Arthralgia/arthritis ■ Maculopapular rash □ Significant weight loss □ Loss of appetite □ Lymphadenopathy □ Macrophage activation syndrome □ Diffuse edema □ Splenomegaly □ Headache □ Pleurisy □ General weakness □ Chest pain	■ Fever ■ Sore throat □ Myalgia ■ Arthralgia/arthritis □ Maculopapular rash □ Significant weight loss □ Loss of appetite ■ Lymphadenopathy (paraaortic and aortocaval spaces) □ Macrophage activation syndrome ■ Diffuse edema (both hands and feet) ■ Splenomegaly □ Headache □ Pleurisy □ General weakness □ Chest pain	■ Fever ■ Sore throat ■ Myalgia ■ Arthralgia/arthritis ■ Maculopapular rash (upper back, dorsal aspect of the hands, and bilateral thighs) □ Significant weight loss □ Loss of appetite □ Lymphadenopathy □ Macrophage activation syndrome □ Diffuse edema □ Splenomegaly ■ Headache ■ Pleurisy □ General weakness □ Chest pain	■ Fever ■ Sore throat □ Myalgia ■ Arthralgia/arthritis ■ Maculopapular rash (trunk and four limbs, and also on the right palm, sparing the face, without itch) □ Significant weight loss □ Loss of appetite □ Lymphadenopathy □ Macrophage activation syndrome □ Diffuse edema □ Splenomegaly □ Headache □ Pleurisy ■ General weakness □ Chest pain
	Case 2: ■ Fever (quotidian) □ Sore throat □ Myalgia ■ Arthralgia/arthritis □ Maculopapular rash ■ Significant weight loss ■ Loss of appetite □ Lymphadenopathy □ Macrophage activation syndrome □ Diffuse edema □ Splenomegaly □ Headache □ Pleurisy □ General weakness □ Chest pain			
	Case 3: ■ Fever ■ Sore throat □ Myalgia ■ Arthralgia/arthritis ■ Maculopapular rash □ Significant weight loss □ Loss of appetite ■ Lymphadenopathy ■ Macrophage activation syndrome □ Diffuse edema □ Splenomegaly □ Headache □ Pleurisy □ General weakness □ Chest pain			
Diagnostic Criteria of AOSD	Cases 1 and 2: Fautrel criteria Case 3: Yamaguchi criteria	Yamaguchi criteria	Yamaguchi criteria	Yamaguchi criteria
Treatment	Case 1: Naproxen	Steroid pulse therapy, intravenous tocilizumab	Prednisolone	Naproxen, methylprednisolone
	Case 2: Naproxen, tocilizumab, and low-dose methotrexate			
	Case 3: Intravenous pulse methylprednisolone + intravenous immunoglobulin, intravenous tocilizumab			
	Case Report: Adult Onset Still’s Disease after vaccination against COVID-19 [8]	Adult-Onset Still’s Disease After the ChAdOx1 nCoV-19 Vaccine [9]	Adult-onset Still’s disease following COVID-19 vaccination [10]	
Age of onset (years) and sex	47 (female)	29 (male)	36 (male)	
Onset after vaccination	7 days (1st dose)	2 days (1st dose)	1 day (1st dose)	
Vaccine	ChAdOx1 nCoV-19	ChAdOx1 nCoV-19	ChAdOx1 nCoV-19	
Symptoms	■ Fever ■ Sore throat □ Myalgia ■ Arthralgia/arthritis ■ Maculopapular rash (bilaterally involving thighs, legs, and hands) □ Significant weight loss □ Loss of appetite □ Lymphadenopathy □ Macrophage activation syndrome □ Diffuse edema ■ Splenomegaly □ (hepatosplenomegaly) □ Headache □ Pleurisy □ General weakness □ Chest pain	■ Fever ■ Sore throat ■ Myalgia ■ Arthralgia/arthritis ■ Maculopapular rash (face, trunk and extremities) □ Significant weight loss □ Loss of appetite □ Lymphadenopathy □ Macrophage activation syndrome □ Diffuse edema □ Splenomegaly ■ Headache □ Pleurisy □ General weakness □ Chest pain	■ Fever ■ Sore throat □ Myalgia □ Arthralgia/arthritis ■ Maculopapular rash □ Significant weight loss □ Loss of appetite ■ Lymphadenopathy □ Macrophage activation syndrome □ Diffuse edema ■ Splenomegaly □ Headache □ Pleurisy □ General weakness ■ Chest pain	
Diagnostic Criteria of AOSD	Yamaguchi criteria	Yamaguchi criteria	Yamaguchi criteria	
Treatment	Prednisolone, methotrexate	Methylprednisolone	Methylprednisolone	

## Data Availability

The data sets used and analyzed during the current study are available from the corresponding author upon reasonable request.
